# Anti-fibrotic effect of extracellular vesicles derived from tea leaves in hepatic stellate cells and liver fibrosis mice

**DOI:** 10.3389/fnut.2022.1009139

**Published:** 2022-10-06

**Authors:** Qianyuan Gong, Zhaoyu Zeng, Tao Jiang, Xue Bai, Chunlan Pu, Yaying Hao, Yuanbiao Guo

**Affiliations:** ^1^Medical Research Center, The Third People's Hospital of Chengdu, The Affiliated Hospital of Southwest Jiaotong University, Chengdu, China; ^2^Department of Clinical Laboratory, The Affiliated Hospital of North Sichuan Medical College, Nanchong, China; ^3^Department of Cardiology, The Third People's Hospital of Chengdu, The Affiliated Hospital of Southwest Jiaotong University, Chengdu, China

**Keywords:** hepatic stellate cells (HSCs), liver fibrosis, extracellular vesicles, tea leaves, TGF-β1

## Abstract

**Background:**

Activation of hepatic stellate cells (HSCs) is essential for the pathogenesis of liver fibrosis, there is no effective drug used to prevent or reverse the fibrotic process.

**Methods:**

With human hepatic stellate cell line LX-2 and mouse model of CCl_4_-induced liver fibrosis, we investigated the anti-fibrotic effect to liver fibrosis of extracellular vesicles (EVs) extracted from tea leaves through cytological tests such as cell proliferation, cell migration, and cell fibrotic marker.

**Results:**

It was found that tea-derived EVs (TEVs) inhibited HSCs activation. In CCl_4_-induced liver fibrosis model, TEVs treatment can significantly improve the pathological changes of liver tissue, inhibit collagen deposition, reduce the number of lipid droplets in liver tissue, and reduce serum AST and ALT levels. In addition, TEVs inhibited TGF-β1 signaling and miR-44 in TEVs had the potential inhibitory effect on liver fibrosis.

**Conclusions:**

Taken together, our work suggesting that TEVs are novel therapeutic potential for liver fibrosis.

## Introduction

Liver fibrosis refers to excessive pathological deposition process of the extracellular matrix (ECM) in the liver (1). Liver injured by viruses, alcohol and chemical poisons may lead to occurrence of liver fibrosis (2). Different degrees of liver fibrosis are found in most of 300 million chronic liver disease patients in China, and about 10% of these patients eventually develop to liver cirrhosis (3).

Hepatic stellate cells (HSCs) are critical resident cells for liver functions. HSCs are activated in response to liver injury and consequently produce the collagen-rich extracellular matrix for development of liver fibrosis (4). Convincing evidences show that activation of HSCs is a major driver of liver fibrosis (5). Therapeutic strategies targeting the activation of HSCs are accordingly considered to be effective for delaying or even reversing the development of liver fibrosis (6).

Tea refers to the leaves and buds of the plant *Camellia sinensis* L. More than 1,400 known compounds are isolated and identified from tea, including primary metabolites proteins, carbohydrates, fats and secondary metabolites in tea trees, such as polyphenols, pigments, theanine, alkaloids, aroma substances, saponin, etc (7). Tea polyphenols have a certain blocking effect on liver fibrosis by anti-oxidation and reducing the level of endotoxin in the body (8). One of the polyphenol components of green tea epigallocatechin-3-gallate can suppress the growth of HSCs, inactivate HSCs into a senescence phenotype, which attenuates production of collagen and collagenase activity (9, 10). Tea flavonoids present a protective effect on liver fibrosis through inflammation and preventing oxidative stress and probably through TGF-β1/Smad signaling pathway (11, 12).

Although many metabolites in tea have protective effects on the liver, it is still a question to explore a better delivery the active ingredients into liver. In recent years, the nanobiotechnology is a prevailing strategy emerging in the delivery of tea components, such as nano-encapsulated tea catechins and polyphenols, which enhance therapeutic efficiency (13–15).

Extracellular vesicles are natural nanoparticles with a diameter of 40–100 nm secreted from cells (16). Studies have shown that plants act on mammalian cells through extracellular vesicles reaching different biological effects. For example, extracellular vesicles derived from grapes and grapefruit can target intestinal cells and play a role in protecting mice from dextran sulfate sodium-induced colitis (17). Also, plant extracellular vesicles have a certain protective effect on liver injury. Oral administered ginger-derived vesicles to normal mice are mainly accumulated in the liver and mesenteric lymph nodes, and inhibit ROS production (18). Alcoholic liver injury mice treated with ginger extracellular vesicles decreased blood triglyceride level and liver lipid droplets, and the weight of the liver indicating the therapeutic potential of ginger extracellular vesicles to alcoholic liver injury. Yet, there is no knowledge about the impact of plant extracellular vesicles on liver fibrosis.

In this study, we found that extracellular vesicles derived from tea leaves (TEVs) inhibited activation of human hepatic stellate cell LX-2 cells and reduced the progression of liver fibrosis in carbon tetrachloride (CCl_4_)-induced mouse fibrosis. Interestingly, miR-44 in TEVs had the potential inhibitory effect on liver fibrosis. Collectively, our data suggest that TEVs may be novel therapeutic biomaterials to prevent HSCs activation and liver fibrosis.

## Methods

### Isolation of TEVs

Fresh leaves from old tea tree (LCC), Fuding tea tree (FD), Meizhan tea tree (MZ) were collected from Chengdu Jiuru village Tea Co., LTD after Tomb Sweeping Day in spring, and the tea leaves are 2–3 young leaves with terminal buds, using cold phosphate buffer saline (PBS) to wash and homogenize tea leaves in a blender. EVs were isolated by a separation method combined differential centrifugation followed with ultrafiltration (19), using Pierce^TM^ BCA Protein Assay Kit (Thermo Fisher Scientific, USA) to determine the protein concentration (20). TEVs were stored at −80°C.

### Particle ultrastructure and size characterization

For ultrastructure inspection by transmission electron microscope (TEM) analysis, TEVs were negatively stained with 2% phosphotungstate solution for 2 min. Imaging was performed by a TEM (JEM-1400, JEOL, Japan).

TEVs were counted by nanoparticle tracking analysis (NTA) (Particle Metrix, Meerbusch, Germany) using a 488 nm laser. The diameter and concentration of extracellular vesicles were detected and analyzed.

### Cellular and tissue uptake assays

LX-2 cells were purchased from Zhong Qiao Xin Zhou Biotechnology Co., Ltd. (Shanghai, China) and seeded on 12-chamber slides (Corning, Kennebunk, USA) at a density of 50,000 cells per well. The next day, TEVs (10 μg/mL) previously stained with DiI (AAT Bioquest, USA) were added to the media for 24 h, then the cells were fixed with 4% paraformaldehyde (Wako, Japan) and nuclei were stained by DAPI (Biosharp, Hefei, China). Finally, the intracellular uptake of TEVs was analyzed using a fluorescence microscope (Leica Microsystems, Wetzlar, Germany).

To study TEVs taken up by liver tissue *in vivo*, healthy mice were orally administered with DiR-loaded TEVs (1 mg protein/kg) for 24 h, mice were sacrificed and liver tissues were fixed in 4% paraformaldehyde.

### Measurement of the growth of LX-2 cells

LX-2 cells were seeded into 96-well-plates at a density of 5,000 cells per well. On Day 0, TGF-β1 (R&D Systems, Minneapolis, USA) (1 ng/mL) or Recombinant Human PDGF-AA (Peprotech, Cranbury, USA) (10 ng/mL) was added to activate the LX-2 cells for 3 h. Then TEVs (10 μg/mL) were added to activated or non-activated LX-2 cells and incubated for 48 h. The growth of LX-2 cells was measured by Cell Counting Kit-8 (CCK-8) (Biosharp, Hefei, China).

### Scratch wound migration assay

LX-2 cells were seeded into 12-well-plates (10,000 cells/well), using 20 μL pipet tip create a scratch wound crossing through the cell layer. The medium was soon replenished with a new medium containing 8 μM β-D-arabinofuranoside (Ara-C) (Sigma-Aldrich, Shanghai, China) or serum-free for inhibition of cell proliferation. TEVs (10 μg/mL) were added to cells for 24 h. The wound images were taken at 0 and 24 h, using image J software to measure the wound area. The migration area is equal to the change in the wound area over 24 h. The relative migration = Migrated area with TEVs/Migrated area with PBS × 100%.

### Animal studies

To established liver fibrosis model in animal, male C57BL/6J mice with 4 weeks were purchased from the Dossy Experimental Animal Ltd. (Chengdu, China). The mice were intraperitoneally injected with 1% CCl_4_ at a dose of 2 mL/kg in olive oil, twice times a week for 5 weeks. The negative control mice treated with olive oil only.

### AST and ALT measurement

To test for hepatotoxicity, levels of alanine aminotransferase (ALT) and aspartate aminotransferase (AST) activity in serum were measured using an Automatic Biochemical Analyzer (Au5800, Beckman, USA).

### Western blotting

Total proteins from cells or liver tissues were lysed in RIPA buffer, and protein concentration was determined by BCA assay. Proteins were separated by SDS-PAGE and then transferred to a PVDF membrane (Millipore, MA, USA) at 80 V for 2 h.

Membranes were blocked in 5% non-fat milk for 1 h and then incubated with primary antibodies for 16 h with diluted mouse anti-GAPDH (1:5,000; ptm-biolab, Hangzhou, China), rabbit anti-α-SMA (1:1,000, proteintech, Wuhan, China), rabbit anti-COL I (1:1,000, Abcam, USA), mouse anti- SMAD2 (1:1,000, ptm-biolab, Hangzhou, China), SMAD3 mouse monoclonal (1:1,000, proteintech, Wuhan, China). The membranes were incubated with the horseradish peroxidase-conjugated species-specific secondary antibody for 2 h and visualized using chemiluminescence reagent (Millipore, MA, USA).

### Quantification and statistical analysis

The SPSS 16.0 software was used for Student's *t*-test to all data. *P*-value < 0.05 was considered statistically significant. One-way ANOVA used to analyze the differences between individual groups. Data are representative of three independent experiments.

### Immunofluorescence staining and microscopy

For immunofluorescence analysis, cells or liver tissues were fixed in 4% paraformaldehyde (Biosharp, Hefei, China) and blocked with 10% normal goat serum (Solarbio, Beijing, China) prior to incubation with primary antibody overnight at 4°C. Primary antibodies used included: α-SMA rabbit polyclonal (1:100; proteintech, Wuhan, China), COL I rabbit polyclonal (1:150; Abcam, UK). Subsequently, using AF488 or AF594-conjugated secondary antibodies (Invitrogen, USA) to incubate the inserts and using a Leica DM4 B microscope take pictures.

### Real-time PCR assays

Total RNA was extracted from LX-2 cells with Trizol reagent (Life Technologies, Carlsbad, CA, USA). cDNA was prepared using HiScript^®^ III RT SuperMix for qPCR Kit (vazyme, Nanjing, China). Real-time PCR was performed by using ChamQ Universal SYBR qPCR Master Mix (vazyme, Nanjing, China) in a quantitative PCR (qPCR) machine (LightCycler 480 II, Roche, Rotkreuz, Switzerland). Primers used are shown in Supplementary Table 1, using the following conditions: Hold: 94°C for 2 min, followed by 35 cycles at 94°C for 15 s and 58°C for 25 s. The 2^−Δ*ΔCt*^ method was used to analyze relative mRNA abundance, using *GAPDH* as an internal control, using three biological replicates.

### TEVs miRNA library preparation and deep sequencing

The miRNA was extracted from TEVs using HiPure Exosome RNA Kit (Magen, Guangzhou, China). Using 10 ng miRNA to prepare library with the NEXTflex^®^ Small RNA-Seq Kit v3 (bio scientific corporation, TX, USA). In the unbiased reaction, the adaptor was sequentially attached to the 3' and 5' ends of the miRNA. The miRNA with adaptor was reverse transcribed to generate cDNA. The cDNA library construction and library sequencing were performed by China Guangzhou Huayin Health Care Group Co., Ltd. according to the supplier's recommended protocol.

## Results

### Characterization of tea leaves derived EVs

**Figure 1:**
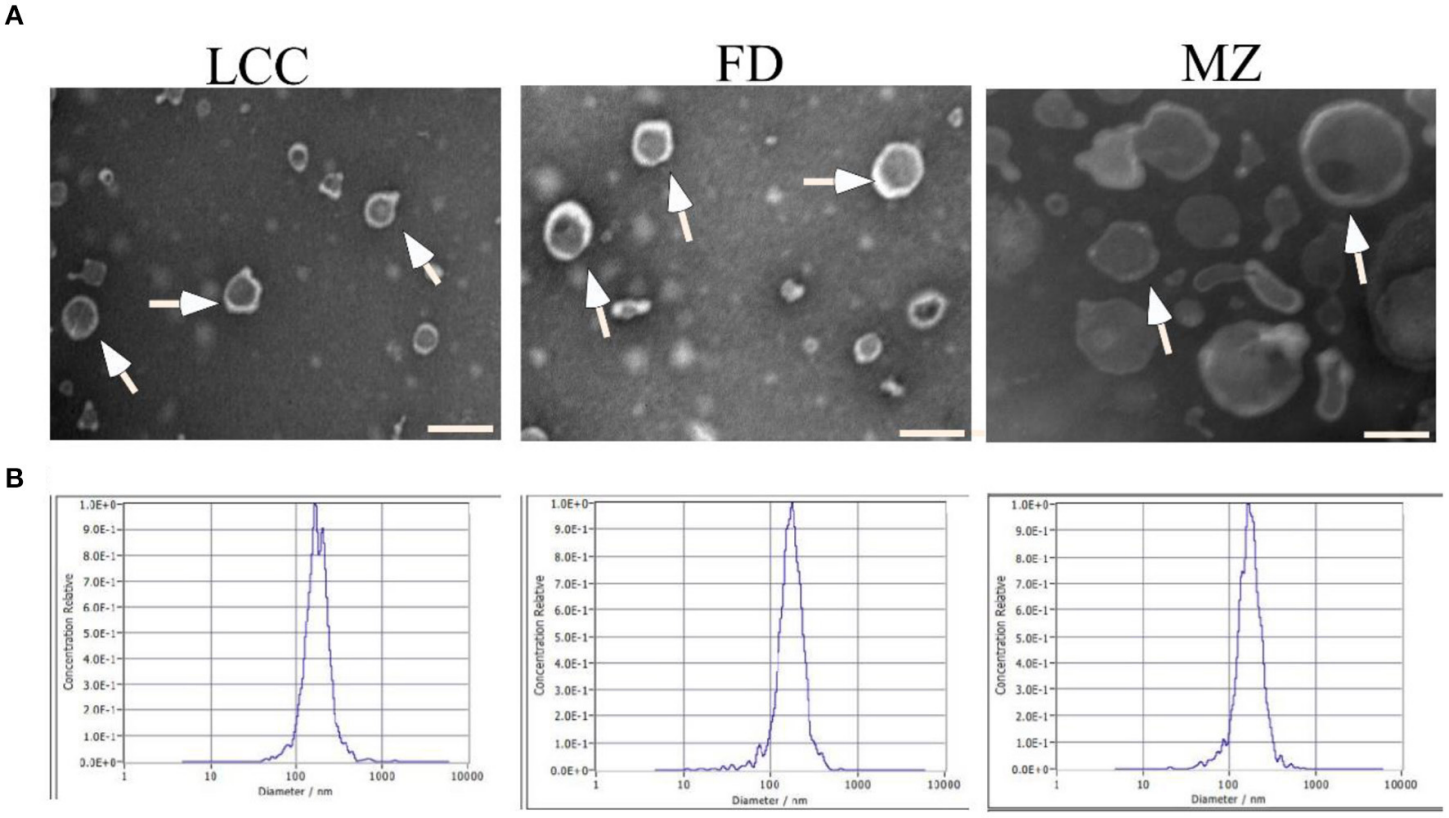
Characterization of EVs from tea leaves. **(A)** TEVs were examined by transmission electron microscopy. Representative TEVs are indicated by white arrows. Scale bar = 200 nm. **(B)** The particle size of TEVs were analyzed by NTA.

To investigate the preventive effect of tea leaf-derived EVs on liver fibrosis, we extracted TEVs from three kinds of tea trees named wild old tea tree (LCC), Fuding (FD), Meizhan (MZ), using a separation method combined differential centrifugation followed with ultrafiltration. TEM revealed that TEVs have a membrane-enclosed vesicle-like structure with size among 50–200 nm (Figure 1A). The size distribution of TEVs was further analyzed by NTA. The average size of TEVs derived from the three trees LCC, FD, and MZ was 172, 169.7, and 169.4 nm, respectively (Figure 1B).

### Cellular uptake of TEVs into liver cells and tissues

**Figure 2:**
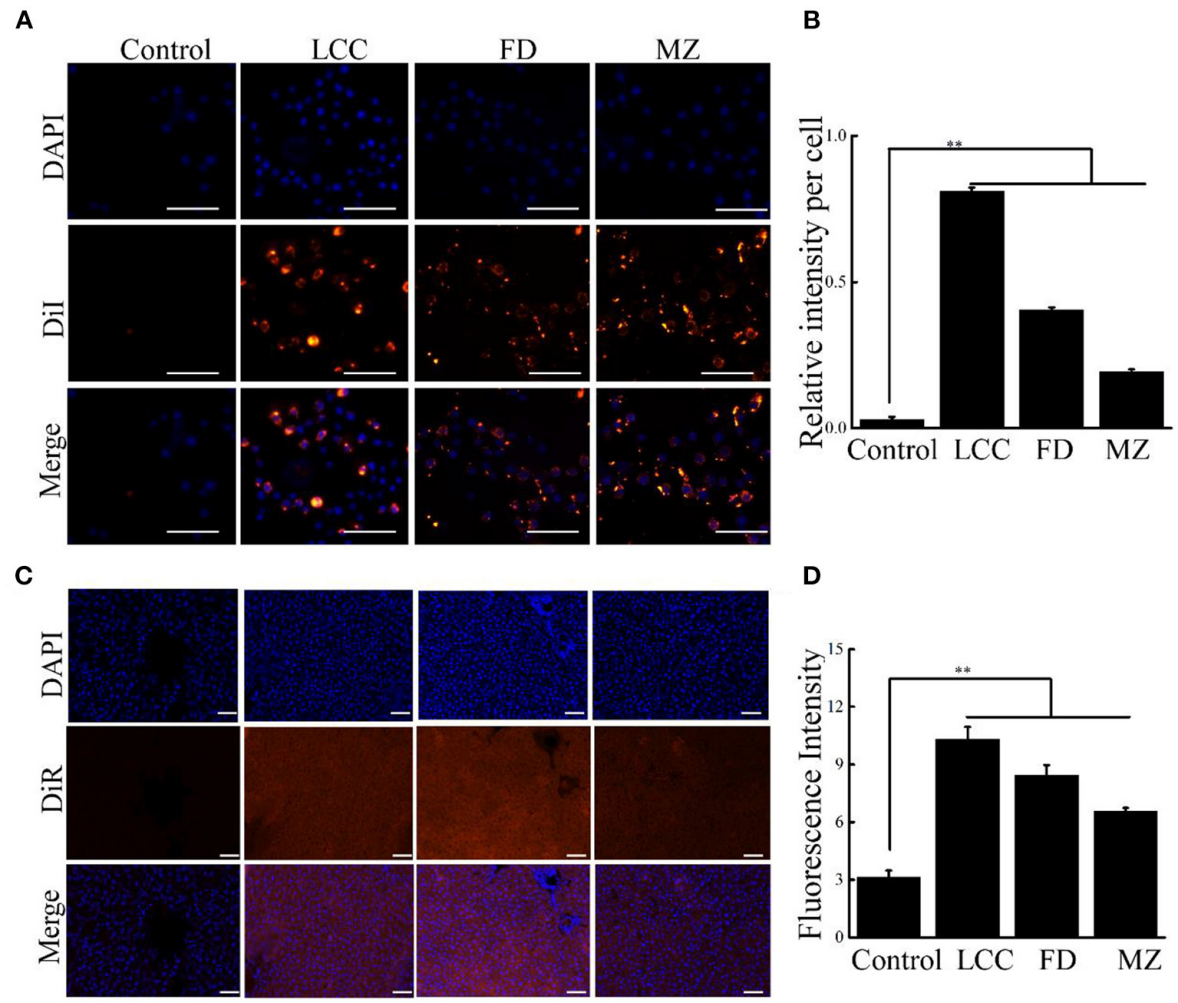
Cellular uptake of TEVs into LX-2 cells and liver tissues. **(A)** Representative fluorescence microscope images of cellular uptake of TEVs at 10 μg/mL. Scale bars = 20 μm. **(B)** Quantification of the uptake of TEVs up to 24 h. **(C)** Fluorescence images showing the accumulation of TEVs in healthy liver tissues after oral administration of DiR-loaded TEVs for 24 h. Scale bars = 50 μm. **(D)** Quantification of fluorescence intensities of the oral administration of DiR-loaded TEVs in mice. Error bar represents SD (*n* = 3; *P < 0.05, **P < 0.01, indicating significantly different from control group).

To probe the effect of TEVs on activation of HSCs, human hepatic stellate cells (LX-2), which are widely used in fibrosis studies, were selected as a cell model (21). After LX-2 cells incubating with TEVs, which were already labeled with DiI, all the three TEVs were found to be enriched in the cell cytosol (Figures 2A,B), indicating that TEVs were successfully up token by LX-2 cells.

As oral administration is a routine delivery for anti-fibrotic therapy. We determine the liver accumulation of oral DiR-loaded TEVs with healthy mice. The fluorescence intensities of liver tissues from DiR-loaded TEVs showed significantly increase compared to the control, while LCC TEVs exhibited the better enrichment than the other two counterparts (Figures 2C,D).

### The effect of TEVs on the proliferation of LX-2 cells

**Figure 3:**
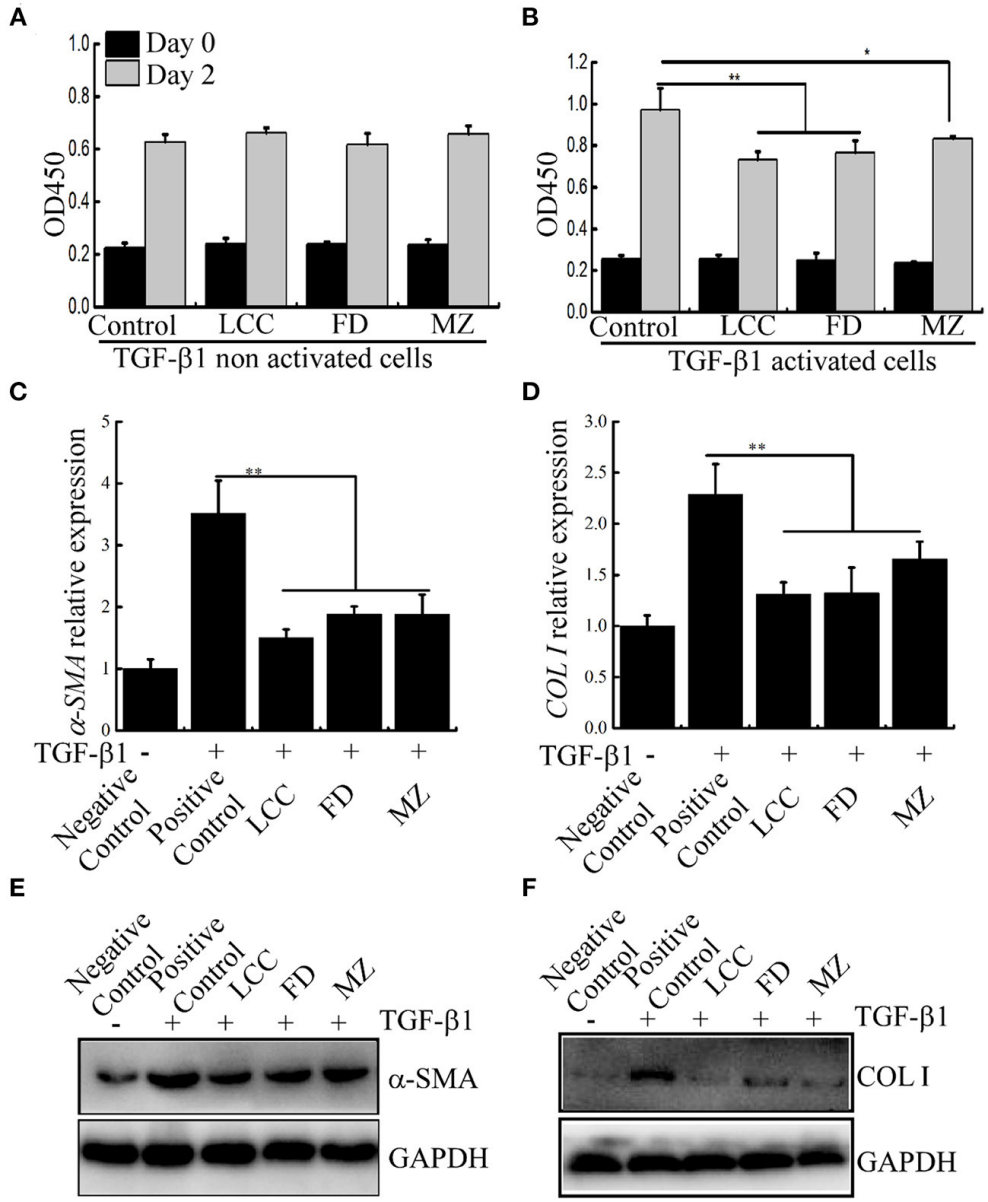
The effects of TEVs on the proliferation of LX-2 cells. **(A)** The effect of TEVs on TGF-β1 non activated LX-2 cells proliferation. **(B)** The effect of TEVs on TGF-β1 activated LX-2 cells proliferation. **(C)** mRNA expression of α*-SMA* was analyzed by Real-time PCR. **(D)** mRNA expression of *COL I* was analyzed by Real-time PCR. **(E)** Protein expression of α-SMA was determined by western blotting. **(F)** Protein expression of COL I was determined by western blotting. Error bar represents SD (*n* = 3; *P < 0.05, **P < 0.01, indicating significantly different from control group).

To observe anti-fibrotic activity of the TEVs, we first examined the effect of TEVs on LX-2 cell proliferation. It showed that TEVs alone did not affect the growth of LX-2 and no cytotoxic effects (Figure 3A). LX-2 cells can be activated from resting (non-activated) state to fibrotic (activated) state by TGF-β1 to initiate the process of liver fibrosis (22). As shown in Figure 3B, LX-2 cells were significantly induced to proliferate after treatment with TGF-β1. After TEVs were added, the proliferation of LX-2 cells was significantly reduced. Dose experiments showed that the inhibitory effect of TEVs on activated LX-2 cells in a dose-dependent manner (Supplementary Figure 1). These results suggest that TEVs can partially counteract the growth trend of LX-2 induced by TGF-β1.

Moreover, we also test this effect of TEVs on another stimulator platelet-derived growth factor (PDGF) for HSCs proliferation (23). However, there is no effect on the proliferation of LX-2 cells (Supplementary Figure 2), indicating that TEVs might not be implicated PDGF signaling pathway in the regulation of cell proliferation.

### The effects of TEVs on the expression of fibrotic markers

COL I and α-SMA are the putative markers were used to evaluate liver fibro (24), were evaluated in LX-2 cells. As shown in Figures 3C–F, after LX-2 cells were treated with TGF-β1, the mRNA and protein expression of marker genes α*-SMA* and *COL I* were significantly up-regulated (about 3.5 times and 2.3 times higher than the control, respectively). In the presence of TEVs, the mRNA level of these two marker scaled downs. Among the three TEVs, TEVs of LCC had the strongest inhibitory effect.

The protein level of α-SMA in LX-2 cells was also evaluated by immunofluorescence staining. TGF-β1 treatment can significantly increase the protein level of α-SMA around the nucleus, while after TEVs treatment, the fluorescence of α-SMA was decreased. In consistence to the mRNA level, LCC TEVs diminished more fluorescence than the others (Supplementary Figure 3). These results implied that TEVs could inhibit the expression of fibrotic marker at both gene and protein levels, suggesting an anti-fibrotic activity.

### TEVs reduced migration of LX-2 cells

**Figure 4:**
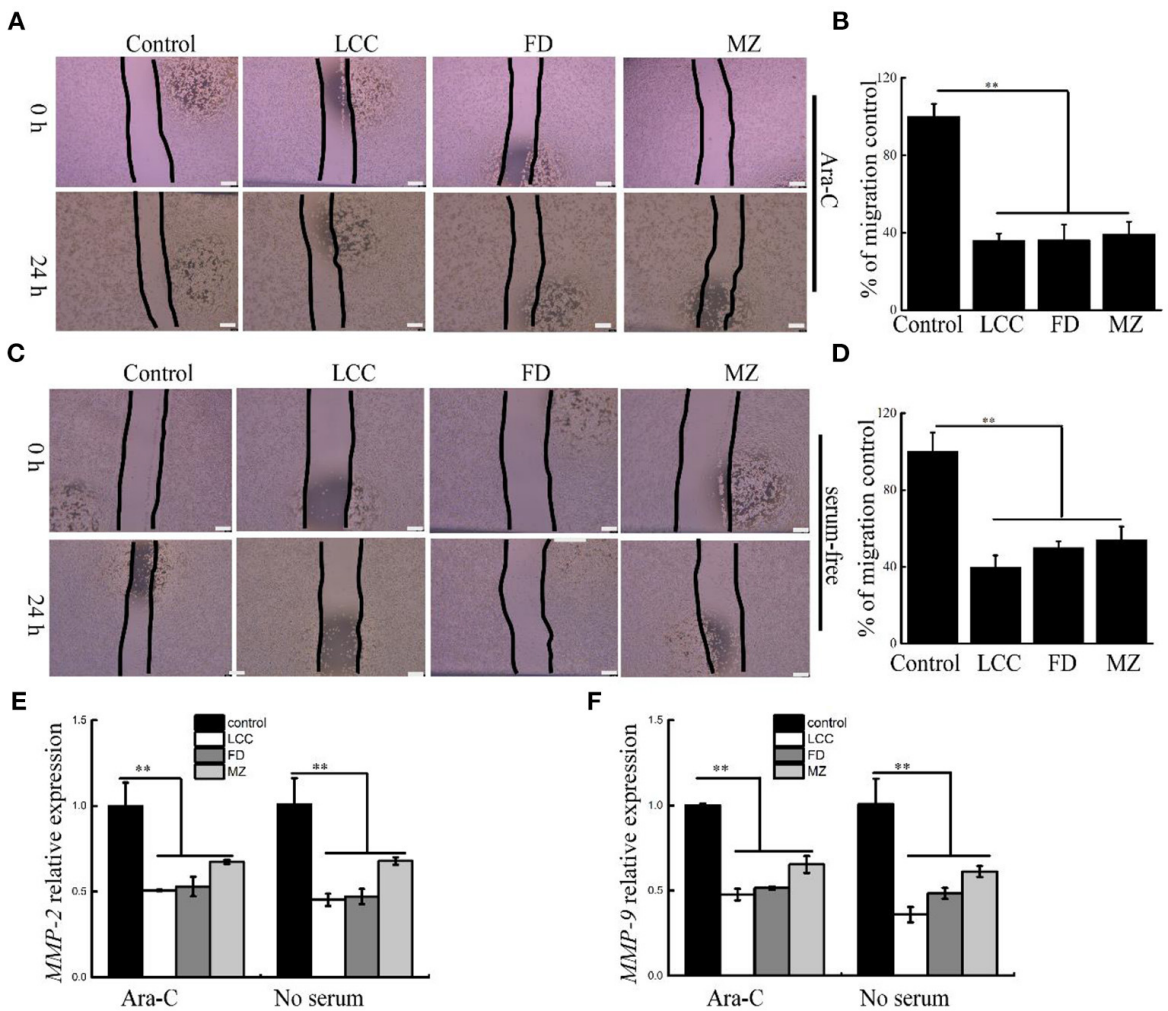
The effects of TEVs on the migration of LX-2 cells. **(A)** Representative images were shown and the edges of the wounds are marked by black lines after treatment with Ara-C and TEVs in a scratch wound assay. Scale bar = 100 μm. **(B)** The area of each wound were quantified in **(A)**. **(C)** Representative images were shown and the edges of the wounds are marked by black lines after treatment with serum-free and TEVs in a scratch wound assay. Scale bar=100 μm. **(D)** The area of each wound were quantified in **(C)**. **(E)** mRNA expression of *MMP-2* was analyzed by Real-time PCR. **(F)** mRNA expression of *MMP-9* was analyzed by Real-time PCR. Error bar represents SD (*n* = 3; *P < 0.05, **P < 0.01, indicating significantly different from control group).

The progression of fibrosis is tightly associated with enhanced migration of LX-2 cells (21). To evaluate inhibition activity of TEVs to cell motility, scratch wound assay was used to assess the migration of LX-2 cells. At the same time, we use Ara-C or serum-free in media to inhibit cell proliferation (25, 26). As shown in Figures 4A–D, TEVs can significantly reduce the migration of LX-2 cells. The mRNA expression of *MMP-2* and *MMP-9*, which belongs to the matrix metalloproteinase (MMP) family and associated with cell migration and invasion (27), were decreased following TEVs treatment relative to the control (Figures 4E,F). These results suggested that TEVs carry anti-fibrotic activity to inhibit the migration of LX-2 cells.

### TEVs reverse liver fibrosis in mice models

**Figure 5:**
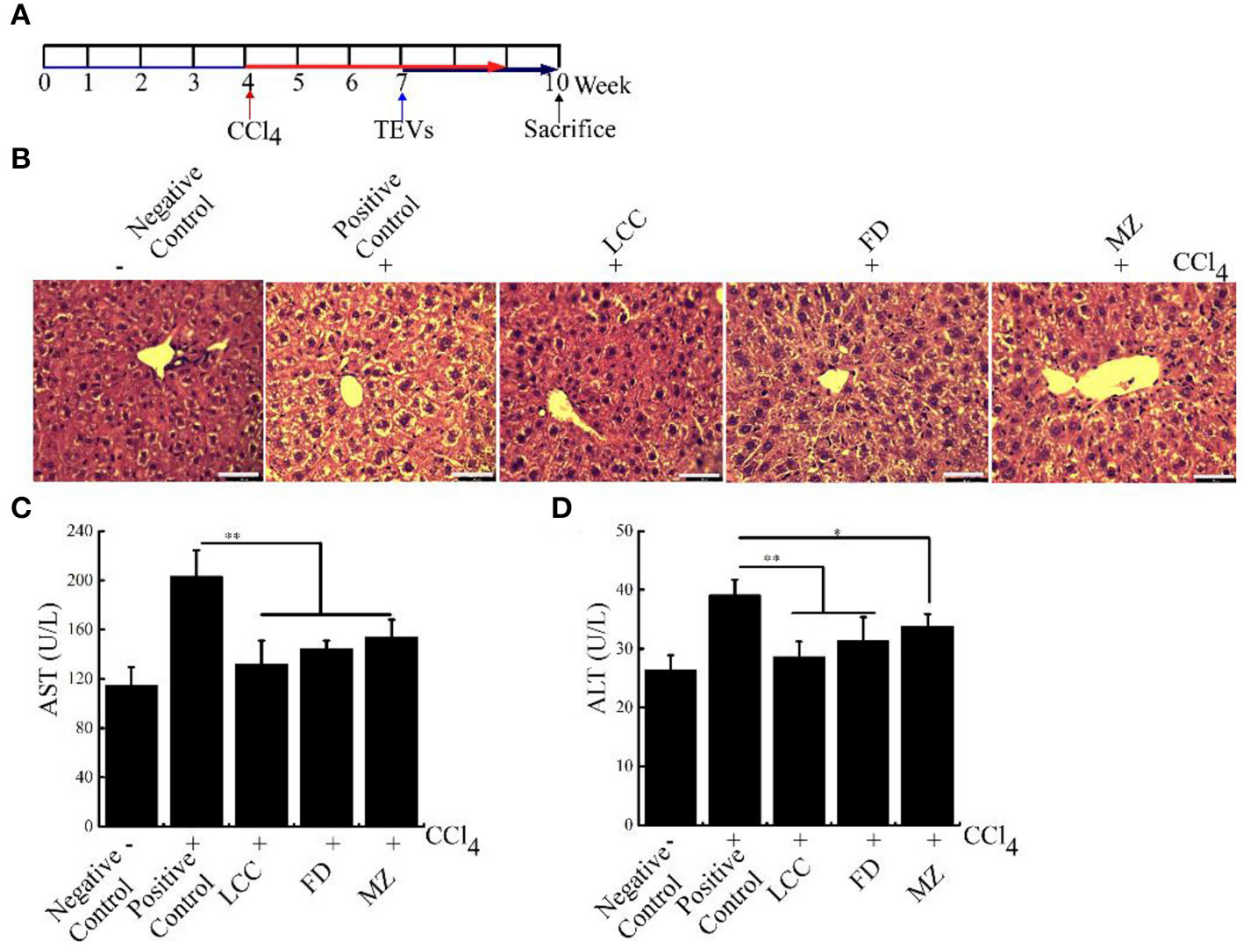
The effects of TEVs on liver fibrosis in mice. **(A)** Flow diagram of experimental design employed in these studies. Mice were injected with 1% CCl_4_ at a dose of 2 mL/kg for twice times a week. On week 7–10, mice were orally administered with various TEVs (1 mg protein/kg mice). **(B)** H&E staining showed that the fibrogenic changes in mice liver. **(C)** Determination of the serum AST activity in the different experimental groups. **(D)** Determination of the serum ALT activity in the different experimental groups. Error bar represents SD (*n* = 3; *P < 0.05, **P < 0.01, indicating significantly different from positive control group).

To further check the therapeutic potential of TEVs against liver fibrosis, a liver fibrosis model was established (Figure 5A). As visualized by H&E staining, CCl_4_ treatment resulted in ECM deposition and led to liver fibrosis (Figure 5B). Furthermore, AST and ALT levels in bloodstream were significantly increased in the positive control group (Figures 5C,D). Compared with the positive control group, the TEVs treatment group showed a significant reduction in ECM deposition and less AST and ALT levels, with the best inhibitory effect showed in the LCC TEVs group (Figure 5).

**Figure 6:**
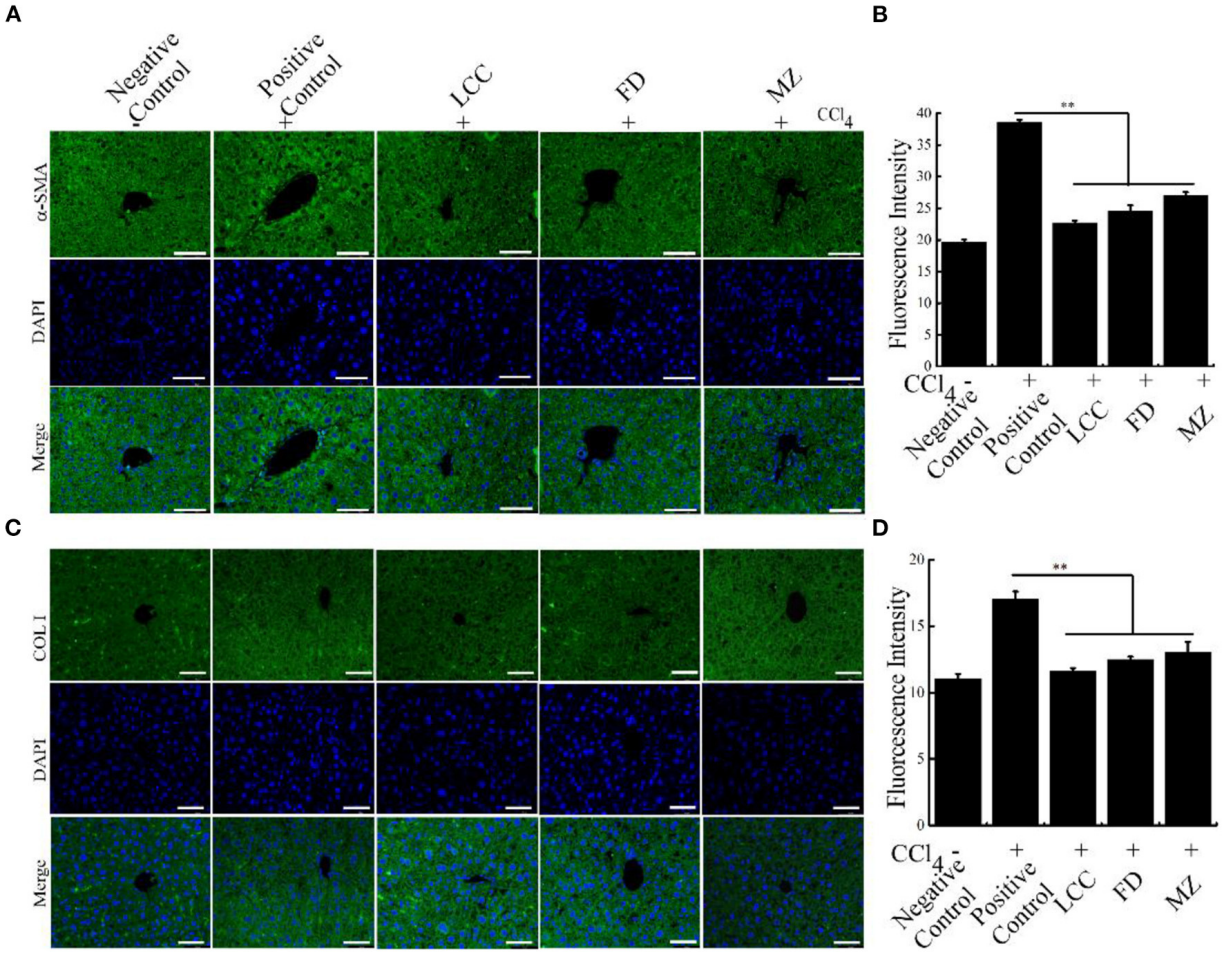
The effects of TEVs on the expression of fibrotic markers in mice. **(A)** Immunofluorescence detection α-SMA protein in the different experimental groups. Scale bar = 50 μm. **(B)** The relative fluorescence intensity in **(A)** was quantified using Image J software. **(C)** Immunofluorescence detection COL I protein in the different experimental groups. Scale bar = 50 μm. **(D)** The relative fluorescence intensity in **(C)** was quantified using Image J software. Error bar represents SD (*n* = 3; *P < 0.05, **P < 0.01, indicating significantly different from positive control group).

Furthermore, compared to the positive control group, in the TEVs treatment group, fibrosis markers α-SMA and COL I were significantly curtailed in intrahepatic duct and vessel walls. These data suggested that TEVs reverse liver fibrosis (Figure 6, Supplementary Figure 2). Taken together, oral administration of TEVs is capable to reverse liver fibrosis.

### The effects of TEVs on the TGF-β1/Smads signal pathway in LX-2 cells

**Figure 7:**
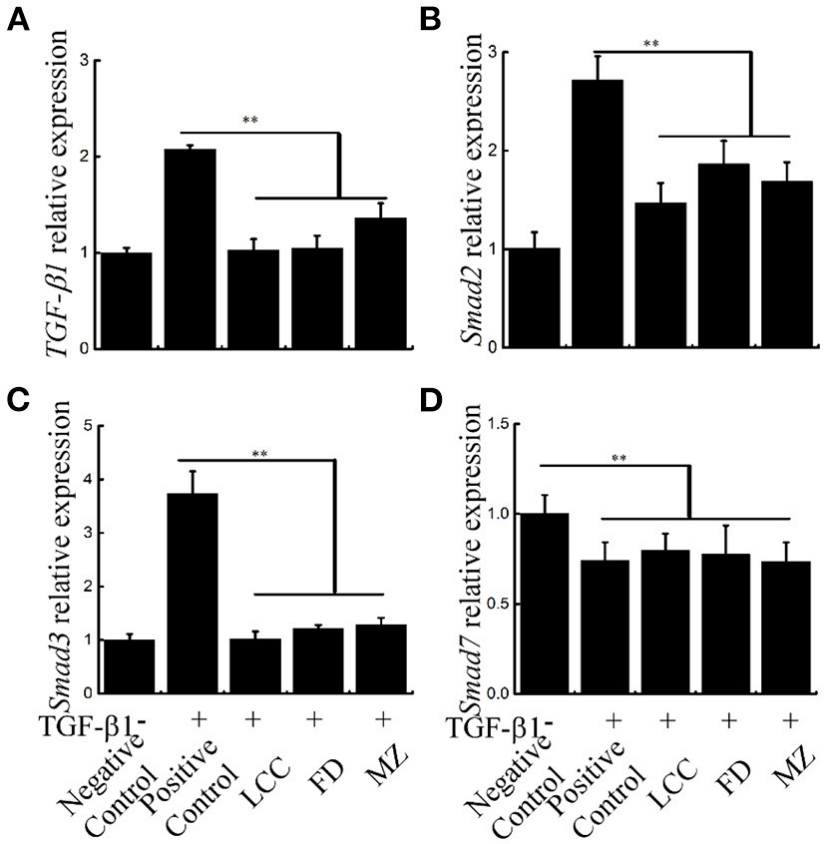
The effects of TEVs on the TGF-β1/Smads signal pathway in LX-2 cells. **(A)** mRNA expression of *TGF-*β*1* was analyzed by Real-time PCR. **(B)** mRNA expression of *Smad2* was analyzed by Real-time PCR. **(C)** mRNA expression of *Smad3* was analyzed by Real-time PCR. **(D)** mRNA expression of *Smad7* was analyzed by Real-time PCR. Error bar represents SD (*n* = 3; *P < 0.05, **P < 0.01, indicating significantly different from control group).

The central link in the formation of liver fibrosis is the activation of HSCs, and TGF-β1-mediated TGF-β1/Smads pathway is the main pathway inducing HSCs activation (28). *TGF-*β*1, Smad2* and *Smad3* mRNA levels were significantly restrained by TEVs treatment relative to the positive control group via Real-time PCR, with the lowest levels seen in the LCC group (Figure 7). These results suggest that TEVs treatment inhibited TGF-β1/Smads pathway.

### The miR-44 in TEVs were identified to inhibit liver fibrosis

**Figure 8:**
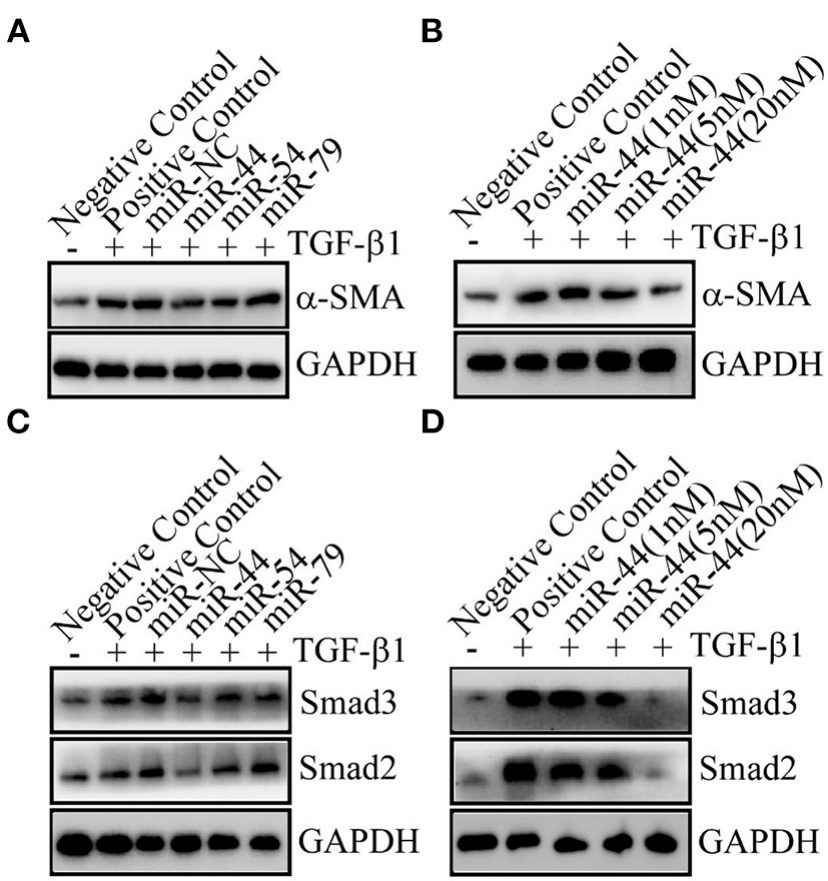
The miR-44 in TEVs was identified to inhibit liver fibrosis. **(A)** The miR-44 can inhibit liver fibrosis. 20 nM of miRNA mimic or miRNA (miR) negative control (NC) were transfected. The protein expression levels of α-SMA were detected. **(B)** The miR-44 dose-dependently decreased α-SMA protein expression. Different amounts of miRNA negative control were co-transfected with miR-44 to ensure the total transfected RNAs of 20 nM. **(C)** The miR-44 had an effect on Smads signal. 20 nM of miRNA mimic or miR-NC were transfected. The protein expression levels of Smad2 and Smad3 were detected. **(D)** The miR-44 dose-dependently decreased Smad2 and Smad3 protein expression. Different amounts of miRNA negative control were co-transfected with miR-44 to ensure the total transfected RNAs of 20 nM.

Among the biocargoes contained in extracellular vesicles, miRNAs have attracted the most interest in terms of their functional and therapeutic importance. Previous studies have shown that the microRNAs (miRNAs) from extracellular vesicles-like nanoparticles of ginger may inhibit the expression of SARS-CoV-2 genes without inducing side effects (29). When humans drink natural edible plant juice, miRNAs (packed in EVs to protect them from degradation) pass through the gastrointestinal tract and are absorbed by small intestine enterocytes (30). In this study, we identified 65 and 63 novel miRNAs with a length distribution of 18–24 nt in LCC and MZ TEVs, respectively (Supplementary Table 2). In view of LCC TEVs showed more therapeutic efficacy than MZ TEVs, among the most abundant miRNAs, miR-44 and miR-54 showed more abundant in LCC TEVs than MZ TEVs, and miR-79 showed higher abundance difference in MZ TEVs than LCC TEVs, so these three miRNAs were selected to synthesize their mimics. These mimics were transfected into LX-2 cells, followed by TGF-β1 induced liver fibrosis. Interestingly, miR-44 could potently decrease the protein expression levels of α-SMA (Figure 8A). Dose experiments showed that the inhibitory effect of miR-44 on liver fibrosis in a dose-dependent manner (Figure 8B). As expected, there were remarkable decreased expression of Smad2 and Smad3 in miR-44 treatment group in a dose-dependent manner (Figures 8C,D). Collectively, these findings indicate that miR-44 in TEVs had the potential inhibitory effect on liver fibrosis.

## Discussion

Liver fibrosis is a wound-healing response to liver cellular damage and is characterized by the deposition of collagen fibers (1, 31). These fibers can go on to cause chronic injury of normal liver, liver cells are replaced by ECM proteins and are accompanied with constant inflammation (32). Following injury, activated HSCs are hyperplastic and contractile, and are characterized by lack of vitamin A storage and glial fibrillary acidic protein expression, high production of α-SMA and secretion of collagens type I (33–37). Therefore, HSCs are the primary target of anti-fibrosis therapy.

In our study, TEVs treatment significantly inhibited the proliferation of LX-2 cells and decreased the fibrosis marker genes expression of α*-SMA* and *COL 1*, with similar results were observed *in vitro* and *in vivo*. In CCl_4_-induced liver fibrosis model, TEVs treatment can significantly improve the pathological changes of liver tissue through inhibit collagen deposition, reduce the number of lipid droplets in liver tissue, and reduce serum AST and ALT levels. In addition, the miR-44 in TEVs had the potential inhibitory effect on liver fibrosis. These results suggest that TEVs can reverse liver fibrosis by suppressing HSCs activation and ECM production.

The miRNA binding to target genes is the basis of their function. Therefore, we predicted the target genes of TEVs miRNA and found that miR-44 likely to regulate genes involved in cell proliferation, such as *signal-induced proliferation-associated 1 like 1* (*Sipal 1*) and *Fibrosin-like 1*, miR-44 may also regulate cytokines interleukin-12 and interleukin-21, which are involved in inflammatory responses (38–41) (Supplementary Table 3). The regulation of cell proliferation related genes by miR-44 is consistent with the effect of TEVs on LX-2 cell growth induced by TGF-β1. Moreover, the progression of liver fibrosis is closely related to the inflammatory process of liver macrophages (42). The verification and regulatory mechanism of miR-44 on liver fibrosis-related target genes need to be further studied in future.

The TGF-β1/Smads pathway is the main pathway in HSCs activation and the progression of liver fibrosis (28). Smad2 and Smad3, as downstream signaling factors of TGF-β1 signaling pathway, can activate HSCs and further promote the occurrence and development of liver fibrosis. Therefore, targeting TGF-β1/Smads signaling pathway has become an important direction for the intervention of liver fibrosis. Our results indicate that TEVs can significantly reduce the expression level of TGF-β1, Smad2, and Smad3 during TGF-β1 induced HSCs activation. In addition, the miR-44 in TEVs decreased the expression of Smad2 and Smad3. These findings suggest that TEVs could reverse liver fibrosis by suppressing HSCs activation and inhibiting the TGF-β1 signaling pathway. However, further studies are warranted to investigate whether oral administration of TEVs and their bioactive component miRNAs could be absorbed through the gastrointestinal tract and exert any anti-fibrotic activity in consumers.

## Conclusions

In conclusion, the present study suggested that TEVs not only inhibited HSCs activation *in vitro*, but also alleviated CCl_4_-induced liver fibrosis in mice. TEVs might be a promising candidate to combat liver fibrosis in the future.

## Data availability statement

The raw sequence data reported in this study have been deposited in the Genome Sequence Archive (Genomics, Proteomics & Bioinformatics 2021) in National Genomics Data Center (Nucleic Acids Res 2022), China National Center for Bioinformation/Beijing Institute of Genomics, Chinese Academy of Sciences (GSA: CRA007888) that are publicly accessible at https://ngdc.cncb.ac.cn/gsa.

## Ethics statement

The animal study was reviewed and approved by the Ethics Committee of ChongQing Medical University.

## Author contributions

QG and XB conceived the project. TJ, YH, and QG performed the experiments. CP, ZZ, and YG analyzed the data and contributed to discussion. YG supervised the project. All authors contributed to the article and approved the submitted version.

## Conflict of interest

The authors declare that the research was conducted in the absence of any commercial or financial relationships that could be construed as a potential conflict of interest.

## Publisher's note

All claims expressed in this article are solely those of the authors and do not necessarily represent those of their affiliated organizations, or those of the publisher, the editors and the reviewers. Any product that may be evaluated in this article, or claim that may be made by its manufacturer, is not guaranteed or endorsed by the publisher.

## Abbreviations

ALT: alanine aminotransferase
α-SMA: α-smooth muscle actin
AST: aspartate aminotransferase
CCl_4_: carbon tetrachloride
COL I: type I collagen
ECM: extracellular matrix
TEVs: tea-derived extracellular vesicles
GAPDH: glyceraldehyde-3-phosphate dehydrogenase
HSCs: hepatic stellate cells
NTA: nanoparticle tracking analysis
PDGF: platelet derived growth factor
TEM: transmission electron microscopy
TGF-β1: transforming growth factor-β1.
