# Supplementary material for: Anti-fibrotic effect of extracellular vesicles derived from tea leaves in hepatic stellate cells and liver fibrosis mice
Source: Front Nutr. 2022 Oct 6;9:1009139. doi: 10.3389/fnut.2022.1009139 (PMC9582986; doi:10.3389/fnut.2022.1009139)
Supplement: Supplementary file 1 [file Data_Sheet_1.docx]

**
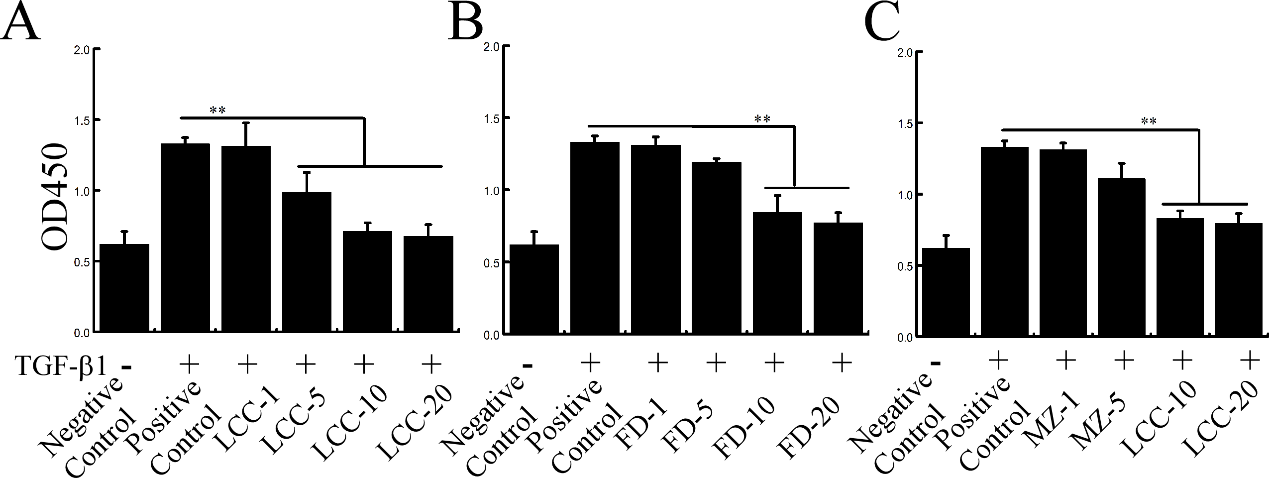
**

**Figure S1. The effects of TEVs on TGF-β1 induced proliferation of LX-2 cells.** The TEVs of LCC (A), FD (B) and MZ (C) dose-dependently reduced the proliferation of LX-2 cells. Different amounts of TEVs (1 µg/5 µg/10 µg/20 µg, represented by LCC/FD/MZ-1/5/10/20) were added to the TGF-β1 activated LX-2 cells for 48 h. The growth of LX-2 cells was measured by CCK-8. Statistical signiﬁcance was determined by a Student’s t-test; signiﬁcant differences are indicated by asterisks (***P* < 0.01).

**
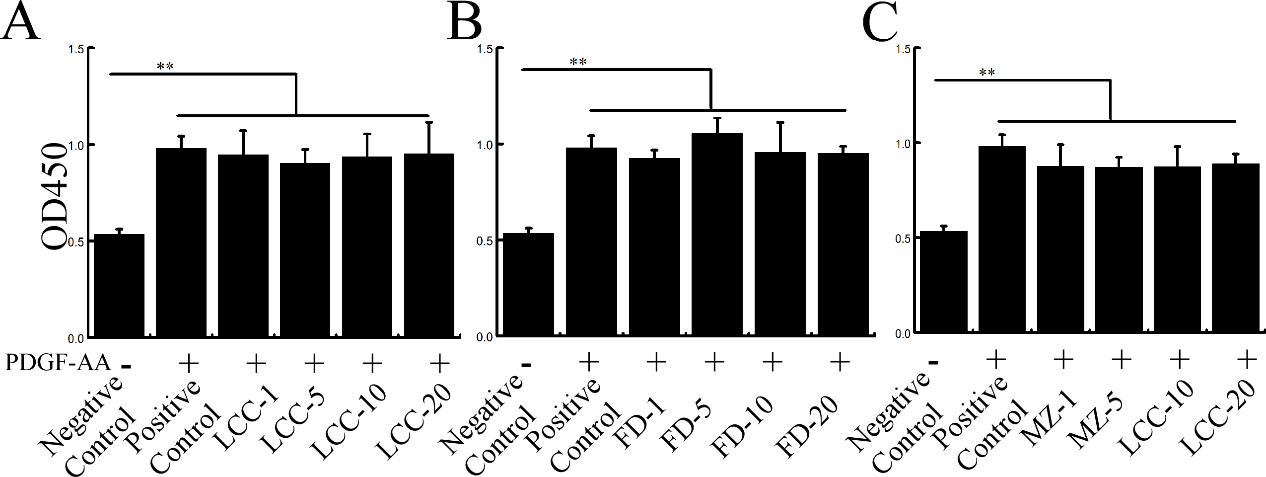
**

**Figure S2. The effects of TEVs on PDGF induced proliferation of LX-2 cells.** The TEVs of LCC (A), FD (B) and MZ (C) dose experiments affect the proliferation of LX-2 cells. Different amounts of TEVs (1 µg/5 µg/10 µg/20 µg, represented by LCC/FD/MZ-1/5/10/20) were added to the PDGF-AA activated LX-2 cells for 48 h. The growth of LX-2 cells was measured by CCK-8. Statistical signiﬁcance was determined by a Student’s t-test; signiﬁcant differences are indicated by asterisks (***P* < 0.01).


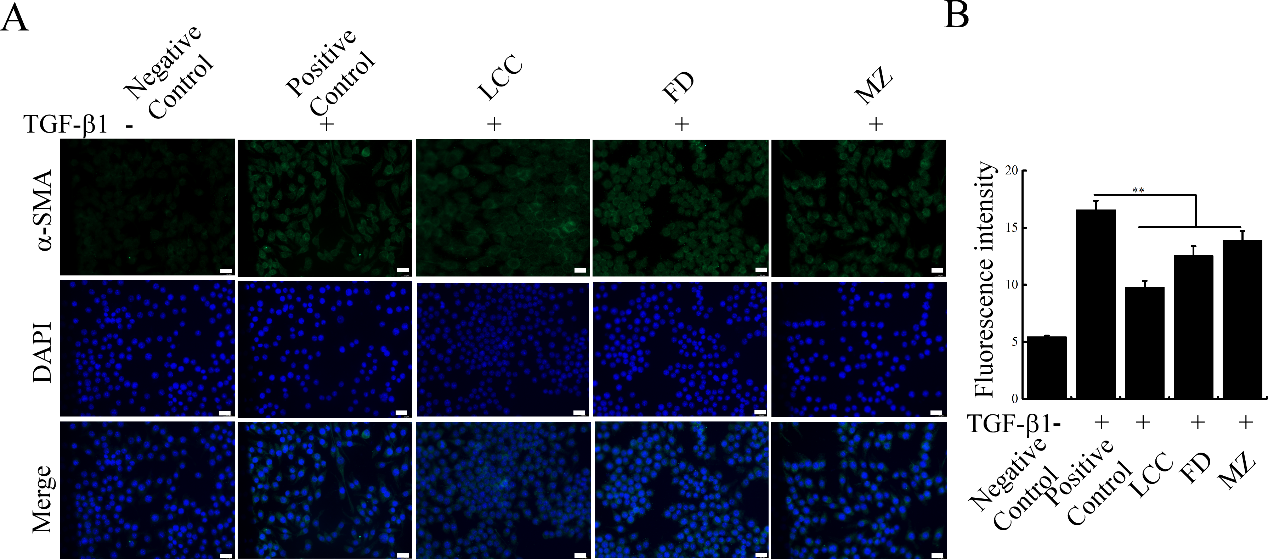


**Figure S3. The effect of TEVs on the protein expression of α-SMA in LX-2 cells.**

(A). Non-activated LX-2 cells were cultured in the presence TEVs for 2 days. Cells were fixed and immunostained with antibody against α-SMA. Representative images are shown. Scale bar = 20 µm.

(B). The relative fluorescence intensity in (A) was quantified using Image J software. Statistical signiﬁcance was determined by a Student’s t-test; signiﬁcant differences are indicated by asterisks (***P* < 0.01).


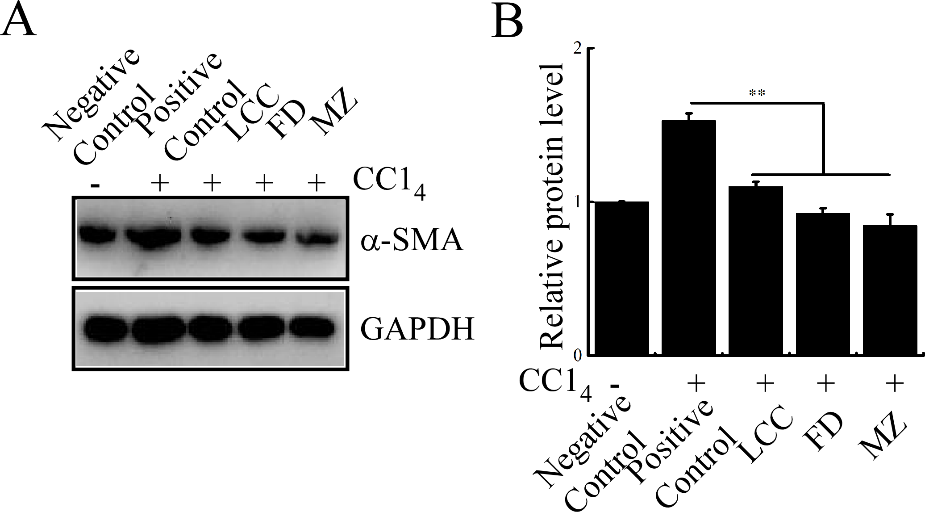


**Figure S4. Effect of TEVs on the protein expression of α-SMA in mice liver.**

(A). Expression levels of α-SMA in the different experimental groups.

(B). Quantification the protein levels of α-SMA in (A). Statistical signiﬁcance was determined by a Student’s t-test; signiﬁcant differences are indicated by asterisks (***P* < 0.01).

**Table S1. Primers used for RT-qPCR assays.**

| Primer name | Sequence from 5’ site to 3’ site |
| --- | --- |
| LP-GAPDH | GACAGTCAGCCGCATCTTCT |
| RP-GAPDH | GCGCCCAATACGACCAAATC |
| LP-TGF-β1 | ATGGAGAGAGGACTGCGGAT |
| RP-TGF-β1 | TAGTGTTCCCCACTGGTCCC |
| LP-Smad2 | GAGCAGAATGGGCAGGAAGA |
| RP-Smad2 | AGAGCAAGTGCTTGGTATGG |
| LP-Smad3 | TGGTACACCGGAAAGCATGG |
| RP-Smad3 | GATTCACGCAGACCTCGTCC |
| LP-Smad7 | AAGTCAAGAGGCTGTGTTGCT |
| RP-Smad7 | CTGGACAGTCTGCAGTTGGTTT |
| LP-COL I | TGATGGGATTCCCTGGACCT |
| RP-COL I | GGGCCTTGTTCACCTCTCTC |
| LP-α-SMA | TTCATCGGGATGGAGTCTGCTGG |
| RP-α-SMA | TCGGTCGGCAATGCCAGGGT |
| LP-MMP-2 | CTGTTGGTGGGAACTCAGAA |
| RP-MMP-2 | CACTTGCGGTCATCATCGTA |
| LP-MMP-9 | GACATCGTCATCCAGTTTGGT |
| RP-MMP-9 | AATGGGCGTCTCCCTGAAT |
